# Supplementary material for: Convergent motifs of early olfactory processing are recapitulated by layer-wise efficient coding
Source: bioRxiv. 2025 Sep 3:2025.09.03.673748. Preprint. [Version 1] doi: 10.1101/2025.09.03.673748 (PMC12424973; doi:10.1101/2025.09.03.673748)
Supplement: 1 [file NIHPP2025.09.03.673748v1-supplement-1.pdf]

bioRxiv preprint doi: https://doi.org/10.1101/2025.09.03.673748; this version posted September 3, 2025. The copyright holder for this preprint (which was not certified by peer review) is the author/funder, who has granted bioRxiv a license to display the preprint in perpetuity. It is made available under aCC-BY 4.0 International license.

Supporting Information

Appendix A: Estimating the minimum effective sensitivity

In our analysis, with the exception of Figure 2, we have initialized the sensitivities W_{ij} to the scale W_{ij}^{min} below which W_{ij} is effectively 0. This allows us to confirm, for example, that broad receptor tuning is not an artifact of initialization (see *SI Appendix: Figs. S2, S6*). Knowing W_{ij}^{min} is also necessary to understand the range of biologically meaningful tuning, which differs from the range of the optimized W_{ij}. For example, many of the optimized elements W_{ij} are far below working machine precision ($\sim 10^{-16}$), but this does not mean that the optimal W spans more than 16 orders of magnitude. Even biologically, for example, $W_{ij} = 10^{-14}$ is equivalent to $W_{ij} = 0$, since none of our odor concentrations are close to 10^{14}. To correctly interpret our results, we must understand the minimum effective value for W_{ij}. We discuss below how that minimum relevant value is set by the maximum concentration c_j.

Assuming canonical expression for simplicity, the mean activity of a neuron expressing the i-th receptor is given by

$$\varphi(W_i^\top c) = \frac{1}{1 + (W_i^\top c)^{-n}}, \tag{A1}$$

where W_i is the vector of sensitivities for the i-th receptor. To determine the minimum relevant sensitivity, we would like to estimate the smallest W_{ij} such that changing the concentration c_j produces a detectable change in $\varphi(W_i^\top c)$. We do so by freezing all components of the concentration vector but the j-th, and write

$$W_i^\top c = a_0 + W_{ij} c_j \quad \text{for} \quad a_0 = \sum_{j' \neq j} W_{ij'} c_{j'}. \tag{A2}$$

Then, we linearize in $W_{ij} c_j$ around the half-occupancy point $a_0 = 1$, which is where the Hill function is maximally sensitive to changes in inputs:

$$\varphi(W_i^\top c) \approx \frac{1}{2} + \frac{n}{4} W_{ij} c_j. \tag{A3}$$

In the absence of neural noise, any contribution above working precision results in an numerically meaningful change in the activity. But for non-zero neural noise levels σ_0, we must estimate a minimum distinguishable difference. For example, in our modeling of the adult fly, $\sigma_0 = 0.1$, and we have approximately 21 neurons per receptor. So the effective noise is approximately $\frac{0.1}{\sqrt{21}}$. Therefore, a rough estimate for a meaningful difference in mean activity compared to the noise is to take $W_{ij} c_j$ to scale with the standard deviation of the effective noise, *i.e.*,

$$W_{ij} c_j \sim \frac{0.1}{\sqrt{21}} \sim \frac{1}{50}. \tag{A4}$$

Other choices for this value simply result in a different prefactor in the final expression for W_{ij}^{min}. To obtain the lowest possible W_{ij}^{min}, we set $c_j = c_{max}$. This results in

$$W_{ij}^{min} = \frac{1}{50 c_{max}}. \tag{A5}$$

This reasoning ensures that $W_{ij} < W_{ij}^{min}$ is functionally equivalent to $W_{ij} = 0$. In other words, the spread of W_{ij} below W_{ij}^{min} is not biologically meaningful. This allows us to make reasonable claims about the distribution of W_{ij}, as in Fig. 2. Finally, recall that c is log-normal, so the maximum c_j is on the order of $c_{max} = \exp(\mu + 3\sigma)$. In this way, the value of W_{ij}^{min} (and thus, the definition of the sparsity of W) depends on the environmental noise.

This argument is purely heuristic, and only intended to guide our interpretation of numerical results. We do not use it as the basis for any biological claims. In particular, we have not conducted any analyses that depend on a precise estimate of W_{ij}^{min}. For example, Qin *et al.* [24] report that as σ_c increases, W_{opt} becomes less sparse (more entries are non-zero). This is plausible, but difficult to confirm in our hands because the meaningful threshold for sparsity is also changing with σ_c. Furthermore, one could take $c_{max} = \exp(\mu + 3\sigma)$, but one might take a more typical value ($\exp(\mu + 2\sigma)$) or a more extreme one ($\exp(\mu + 4\sigma)$). Each choice for c_{max} results in a different W_{ij}^{min}.

bioRxiv preprint doi: https://doi.org/10.1101/2025.09.03.673748; this version posted September 3, 2025. The copyright holder for this preprint (which was not certified by peer review) is the author/funder, who has granted bioRxiv a license to display the preprint in perpetuity. It is made available under aCC-BY 4.0 International license.

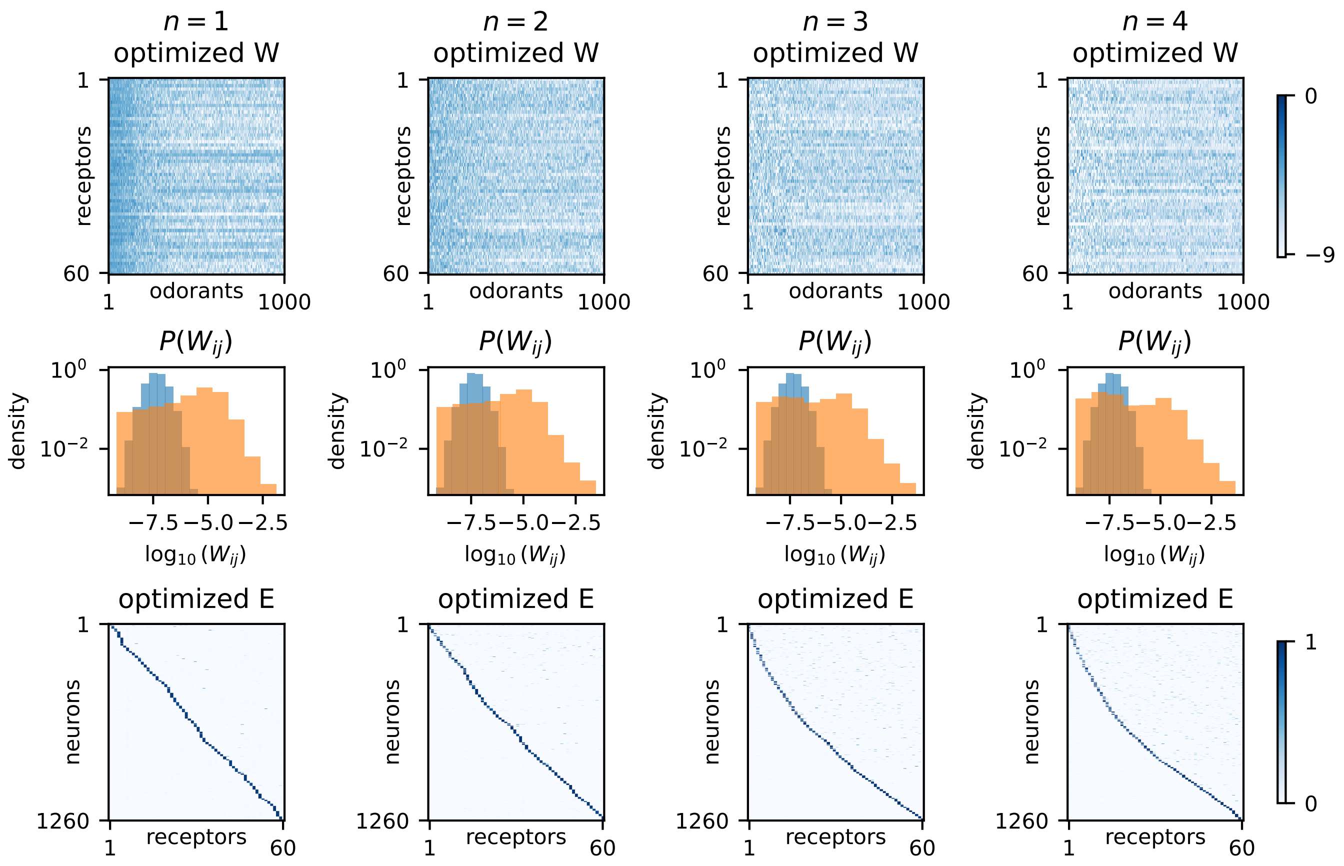

FIG. S1. Effect of the Hill coefficient n on optimized W and E. E optimizations are performed using shuffled W_{opt}. In the histograms, blue represents the initial W_{ij}, and orange the optimized W_{ij}. E was initialized to noncanonical olfaction as in Figure 5a, b.

bioRxiv preprint doi: https://doi.org/10.1101/2025.09.03.673748; this version posted September 3, 2025. The copyright holder for this preprint (which was not certified by peer review) is the author/funder, who has granted bioRxiv a license to display the preprint in perpetuity. It is made available under aCC-BY 4.0 International license.

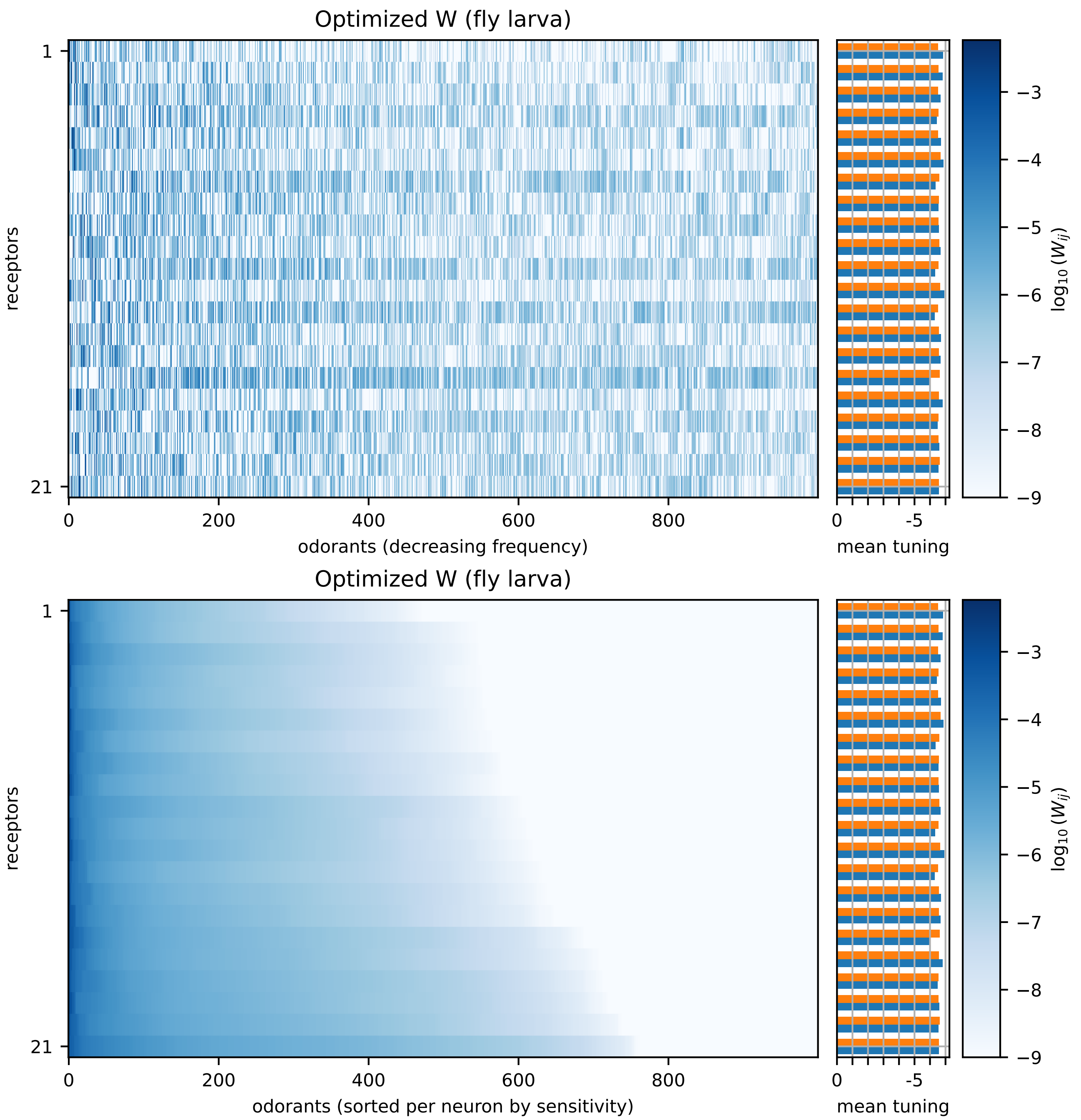

FIG. S2. Receptor tuning curves in the optimized W matrix for the fly larva. The same data is shown twice: for the top panel, odorants are sorted by frequency, whereas for the bottom panel, odorants are sorted within each receptor by sensitivity. Bars on the right indicate the mean log-sensitivity of each receptor (blue is true, orange is shuffled). The receptor mean tuning is more variable than would be expected by chance (blue bars vary more than orange bars). This is consistent with work [11] showing that some receptors are very narrowly tuned (or almost exclusively inhibitory), while others are broadly tuned.

bioRxiv preprint doi: https://doi.org/10.1101/2025.09.03.673748; this version posted September 3, 2025. The copyright holder for this preprint (which was not certified by peer review) is the author/funder, who has granted bioRxiv a license to display the preprint in perpetuity. It is made available under aCC-BY 4.0 International license.

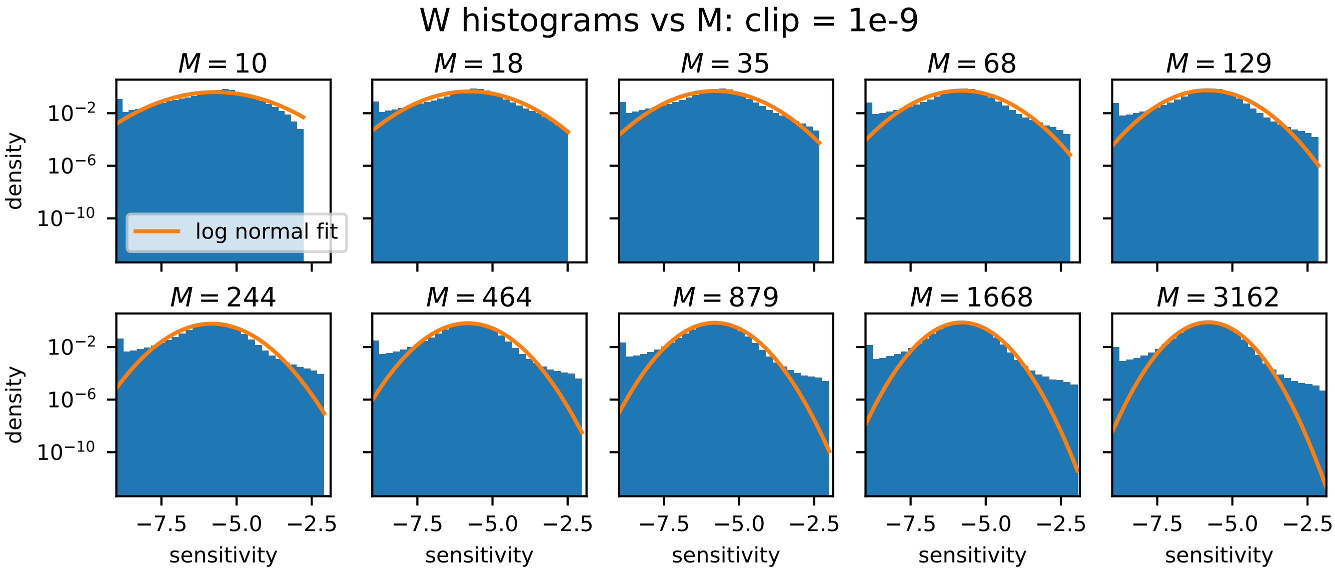

FIG. S3. Histograms of $p(\log_{10}(W_{ij}))$ after optimization, and log-normal fits. Note that the log density is plotted. The heavy right tails of the true distribution compared to the log-normal drive the emergence of specialists for large M (second row).

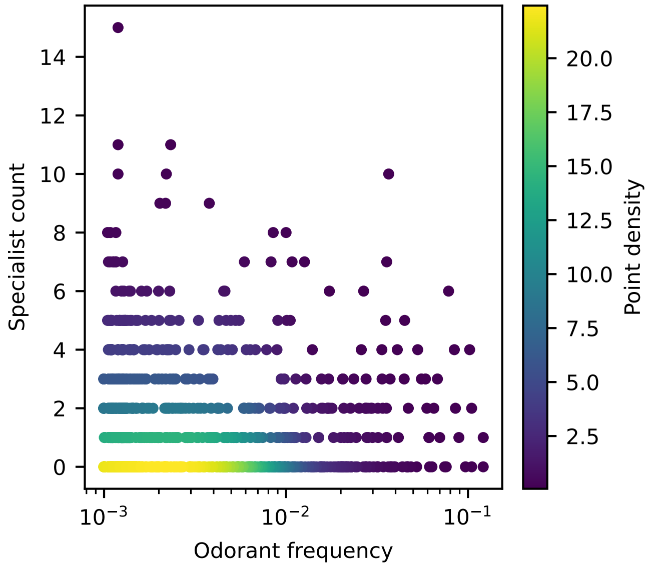

FIG. S4. Specialist receptor count vs. odorant frequency. Specialists targeting higher frequency odorants are not particularly favored.

bioRxiv preprint doi: https://doi.org/10.1101/2025.09.03.673748; this version posted September 3, 2025. The copyright holder for this preprint (which was not certified by peer review) is the author/funder, who has granted bioRxiv a license to display the preprint in perpetuity. It is made available under aCC-BY 4.0 International license.

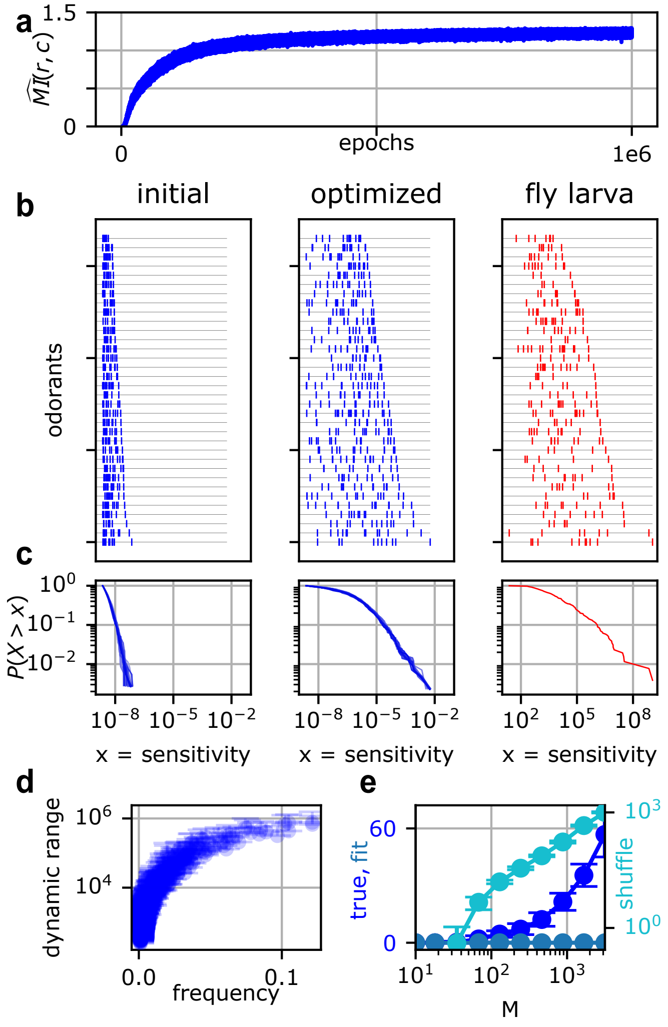

FIG. S5. Optimized W distribution under initialization to minimum W_{ij} (compare to Figure 2 in main text.) The only significant difference is in the "shuffle" curve for panel e, which is shifted dramatically upward (note log scale). This reflects the fact that, for large M, most sensitivities do not need to move under optimization. When W is shuffled, this causes many specialists to emerge. However, in the true W, the number of specialists was comparable to Figure 2e (mean 57 specialists for $M = 3162$ receptors rather than mean 27 specialists for the $1/E[||c||]$ initialization in the main text). This indicates some pressure against developing too many specialists, even in an over-parameterized ($M > N$) regime. See Appendix A for a discussion of how the minimum effective W_{ij} is computed.

bioRxiv preprint doi: https://doi.org/10.1101/2025.09.03.673748; this version posted September 3, 2025. The copyright holder for this preprint (which was not certified by peer review) is the author/funder, who has granted bioRxiv a license to display the preprint in perpetuity. It is made available under aCC-BY 4.0 International license.

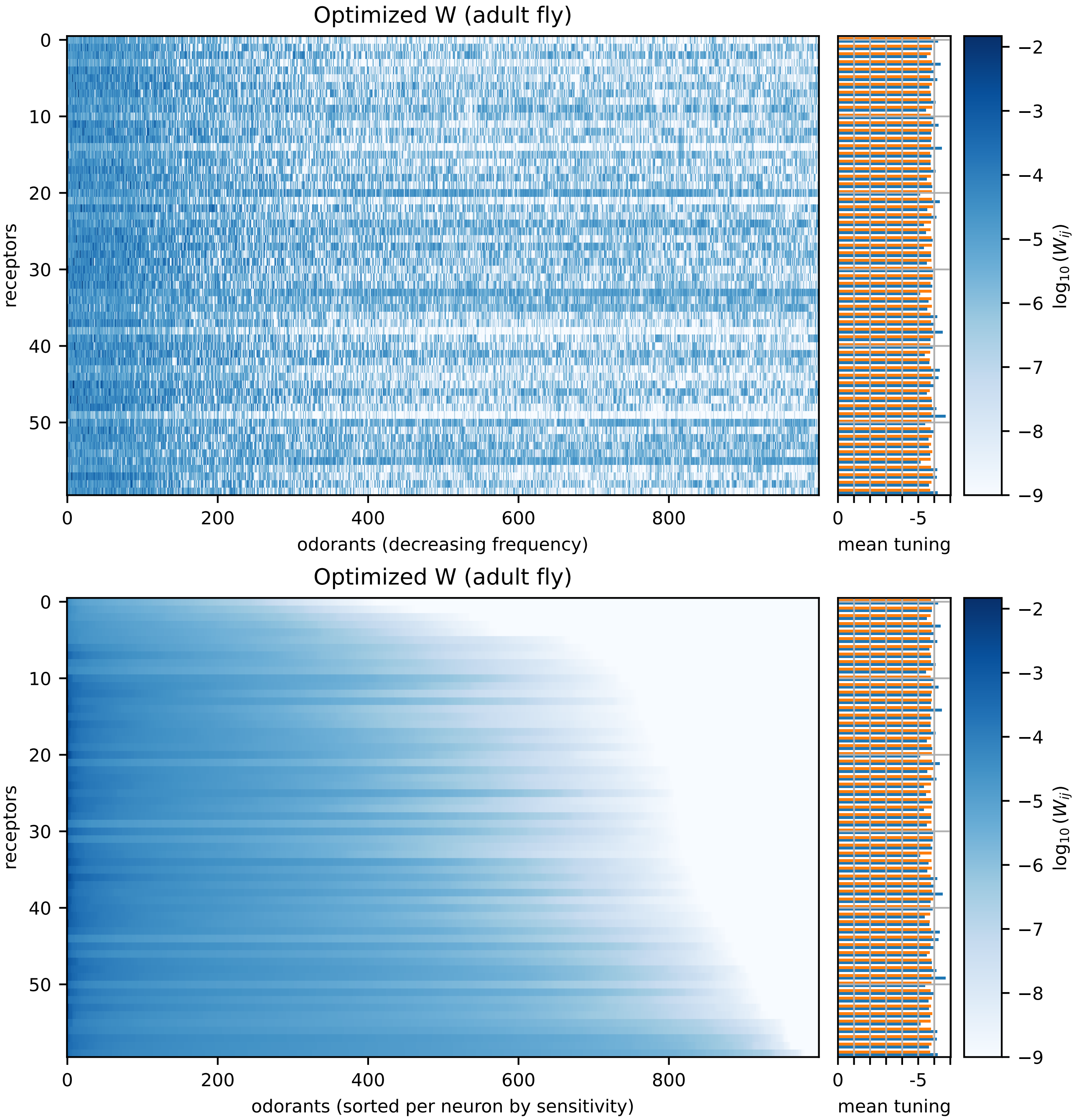

FIG. S6. Receptor tuning curves in the optimized W matrix for the adult fly. The same data is shown twice: for the top panel, odorants are sorted by frequency, whereas for the bottom panel, odorants are sorted within each receptor by sensitivity. Bars on the right indicate the mean log-sensitivity of each receptor (blue is true, orange is shuffled). The receptor mean tuning is more variable than would be expected by chance (blue bars vary more than orange bars). This is consistent with work [11] showing that some receptors are very narrowly tuned (or almost exclusively inhibitory), while others are broadly tuned.

bioRxiv preprint doi: https://doi.org/10.1101/2025.09.03.673748; this version posted September 3, 2025. The copyright holder for this preprint (which was not certified by peer review) is the author/funder, who has granted bioRxiv a license to display the preprint in perpetuity. It is made available under aCC-BY 4.0 International license.

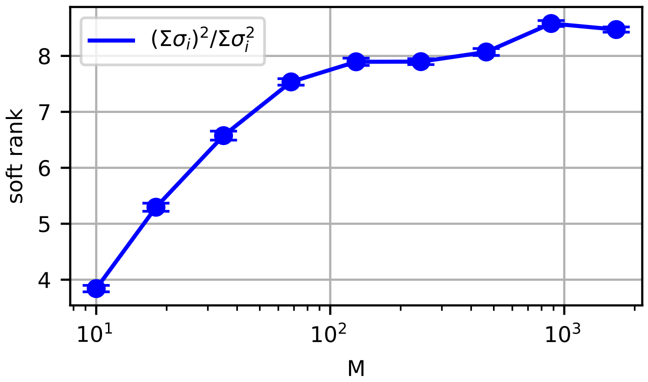

FIG. S7. Soft rank of W vs. receptor count M. The soft rank is computed using $(\sum \sigma_i)^2/\sum(\sigma_i^2)$, where σ_i are the singular values of W. This measure is 1 when W is rank 1 and M when W is full-rank. Error bars indicate the standard deviation over 20 runs.

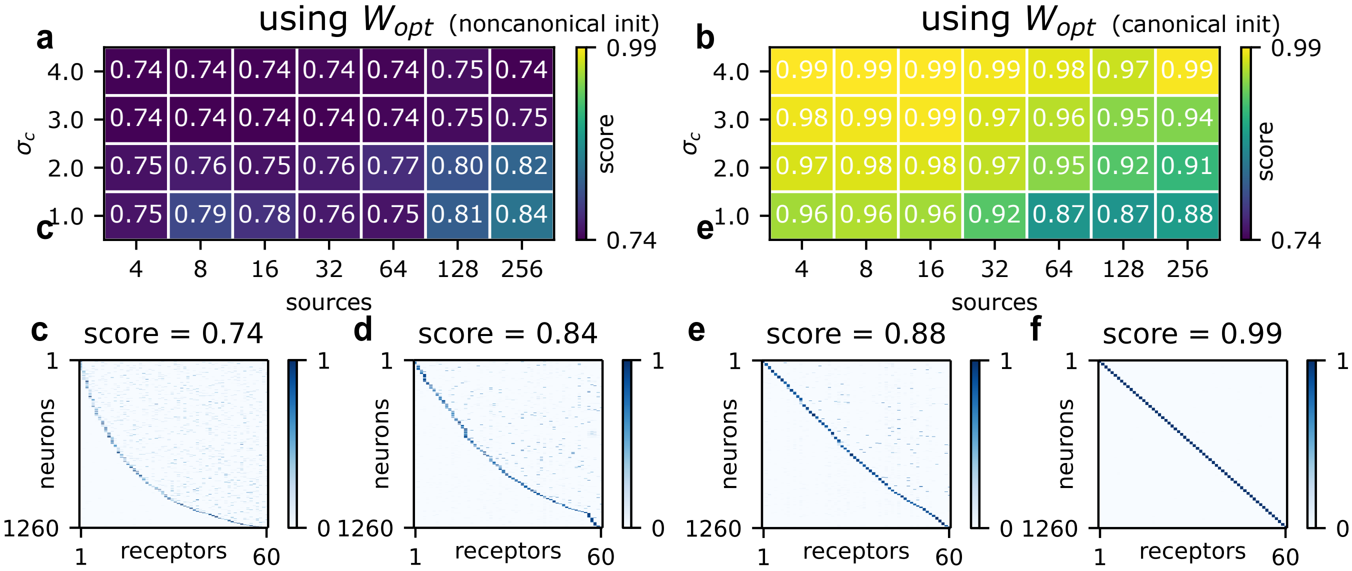

FIG. S8. Phase diagram of E optimization given W_{opt}, comparing canonical and noncanonical initialization. Only the optimizations in the bottom right corner (low noise, many sources) of the phase diagram move meaningfully from initialization.

bioRxiv preprint doi: https://doi.org/10.1101/2025.09.03.673748; this version posted September 3, 2025. The copyright holder for this preprint (which was not certified by peer review) is the author/funder, who has granted bioRxiv a license to display the preprint in perpetuity. It is made available under aCC-BY 4.0 International license.

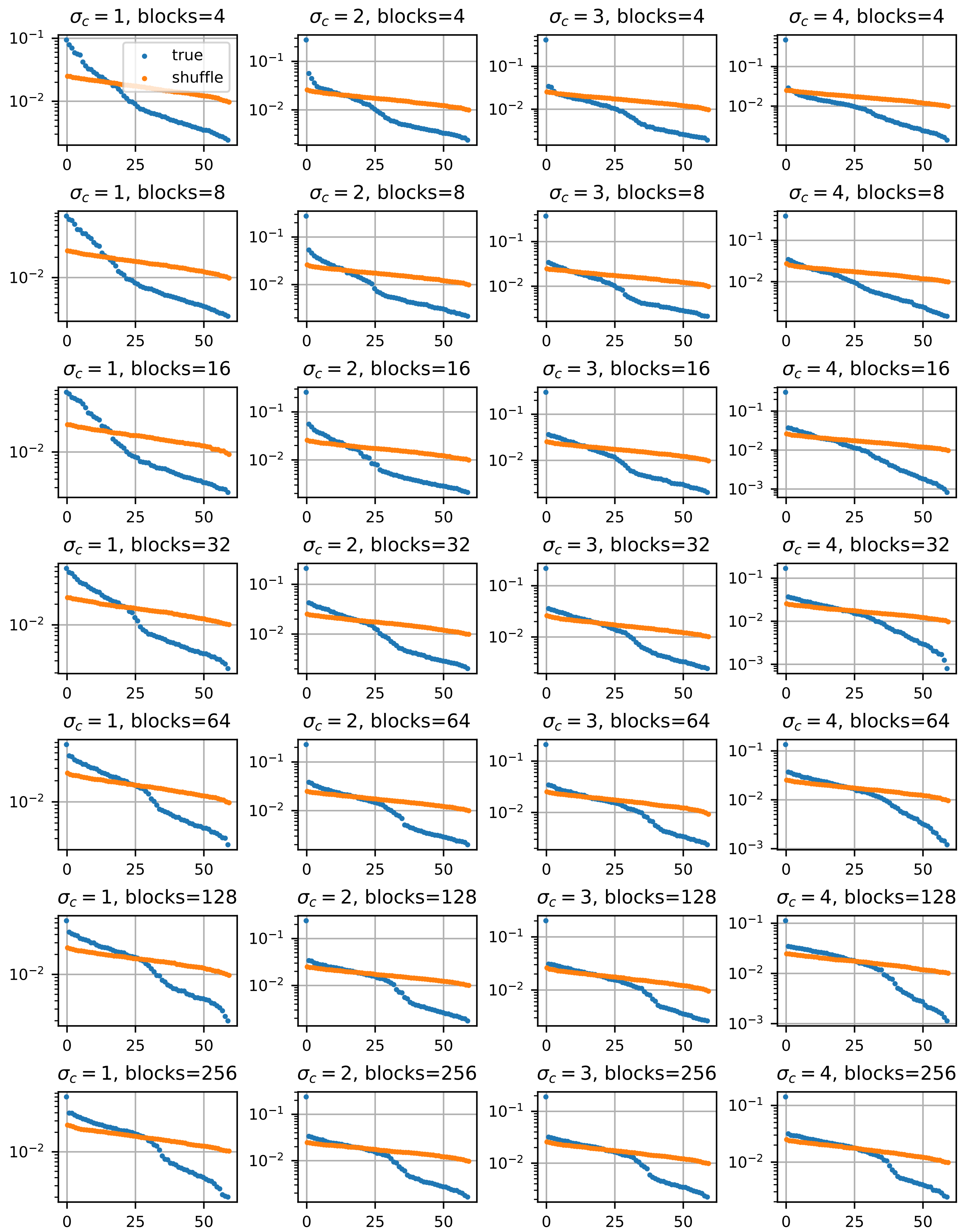

FIG. S9. Eigenvalue spectra of optimized W across environments, given the standard odorant model (most odorants are rare, some are common, as in Figure 1).

bioRxiv preprint doi: https://doi.org/10.1101/2025.09.03.673748; this version posted September 3, 2025. The copyright holder for this preprint (which was not certified by peer review) is the author/funder, who has granted bioRxiv a license to display the preprint in perpetuity. It is made available under aCC-BY 4.0 International license.

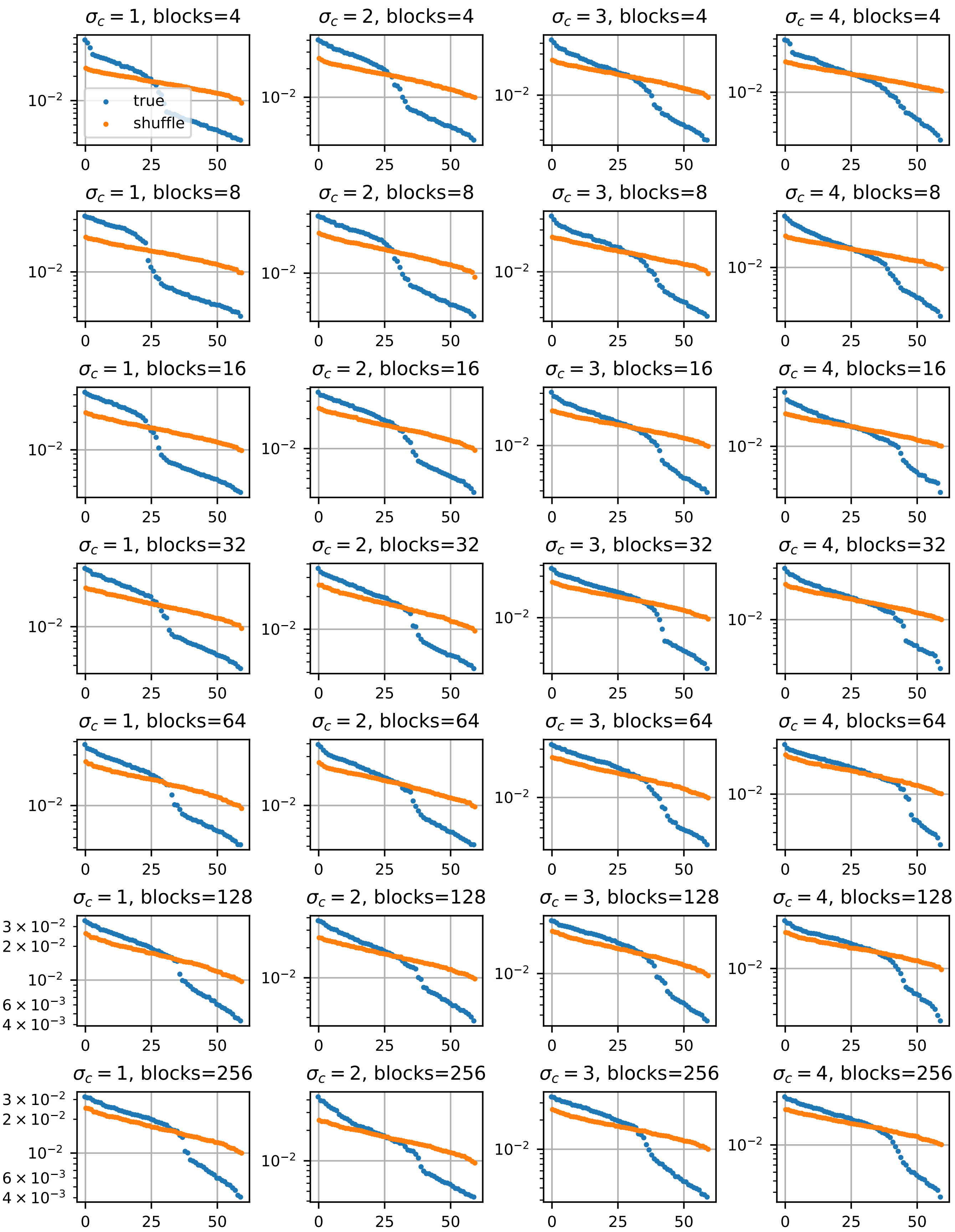

FIG. S10. Eigenvalue spectra of W under an odorant model with constant frequencies (so all $N = 1000$ odorants occur with equal probability). The low rank structure in Fig. S9 is abolished.

bioRxiv preprint doi: https://doi.org/10.1101/2025.09.03.673748; this version posted September 3, 2025. The copyright holder for this preprint (which was not certified by peer review) is the author/funder, who has granted bioRxiv a license to display the preprint in perpetuity. It is made available under aCC-BY 4.0 International license.

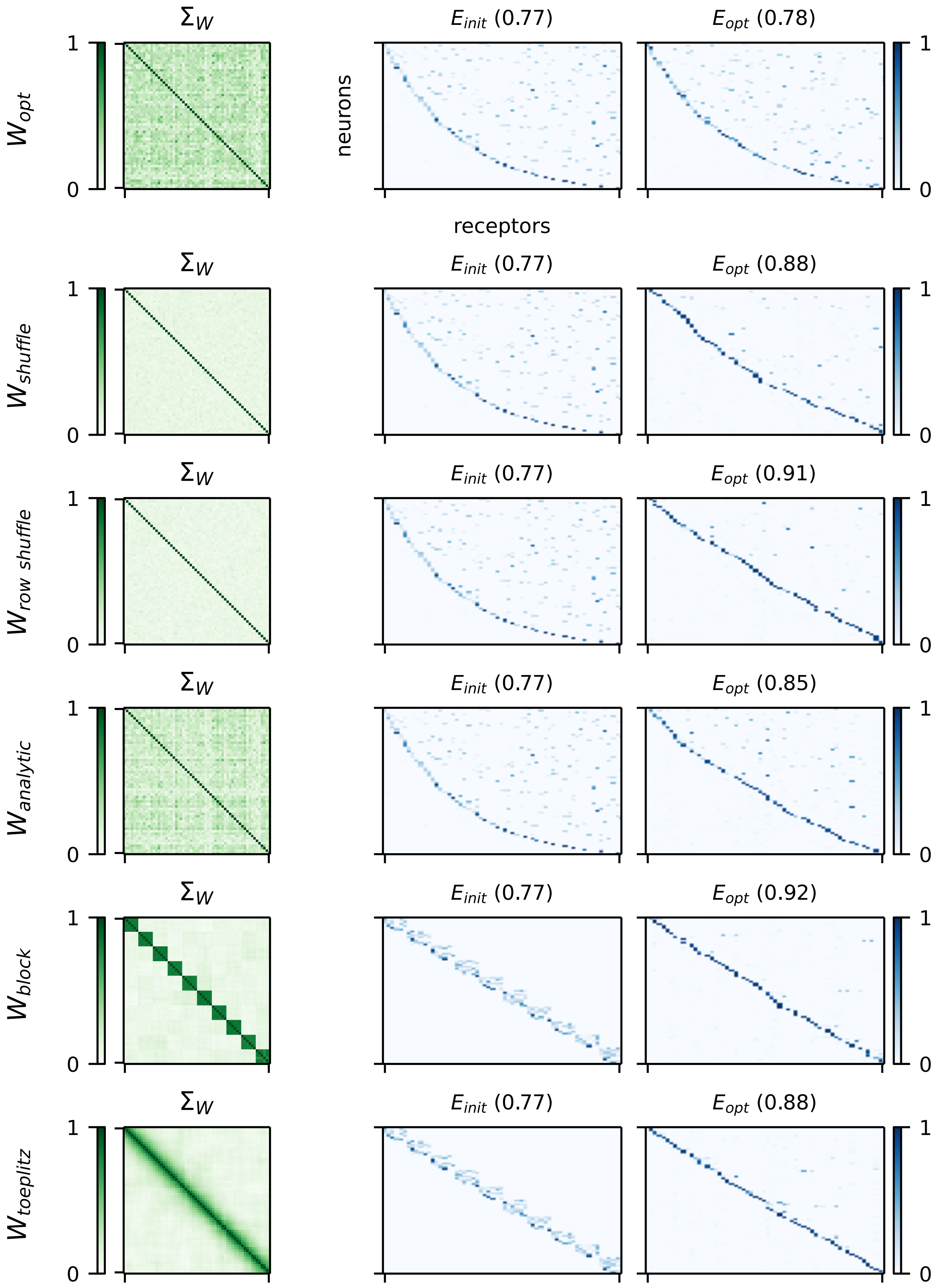

FIG. S11. Optimized expression given a variety of different W matrices. Environmental parameters are set to 64 sources and $\sigma_c = 2$. For W_{block} and $W_{toeplitz}$, the E matrices were initialized to exhibit coexpression of correlated receptors (coexpression by descent) [45]. This did not lead to more noncanonical expression than the shuffled W, in which unrelated receptors were coexpressed. $W_{analytic}$ is generated by fitting a log normal to W_{opt}, then masking by the same sparsity structure present in W_{opt}. This induces a non-negligible correlation structure in $W_{analytic}$.

bioRxiv preprint doi: https://doi.org/10.1101/2025.09.03.673748; this version posted September 3, 2025. The copyright holder for this preprint (which was not certified by peer review) is the author/funder, who has granted bioRxiv a license to display the preprint in perpetuity. It is made available under aCC-BY 4.0 International license.

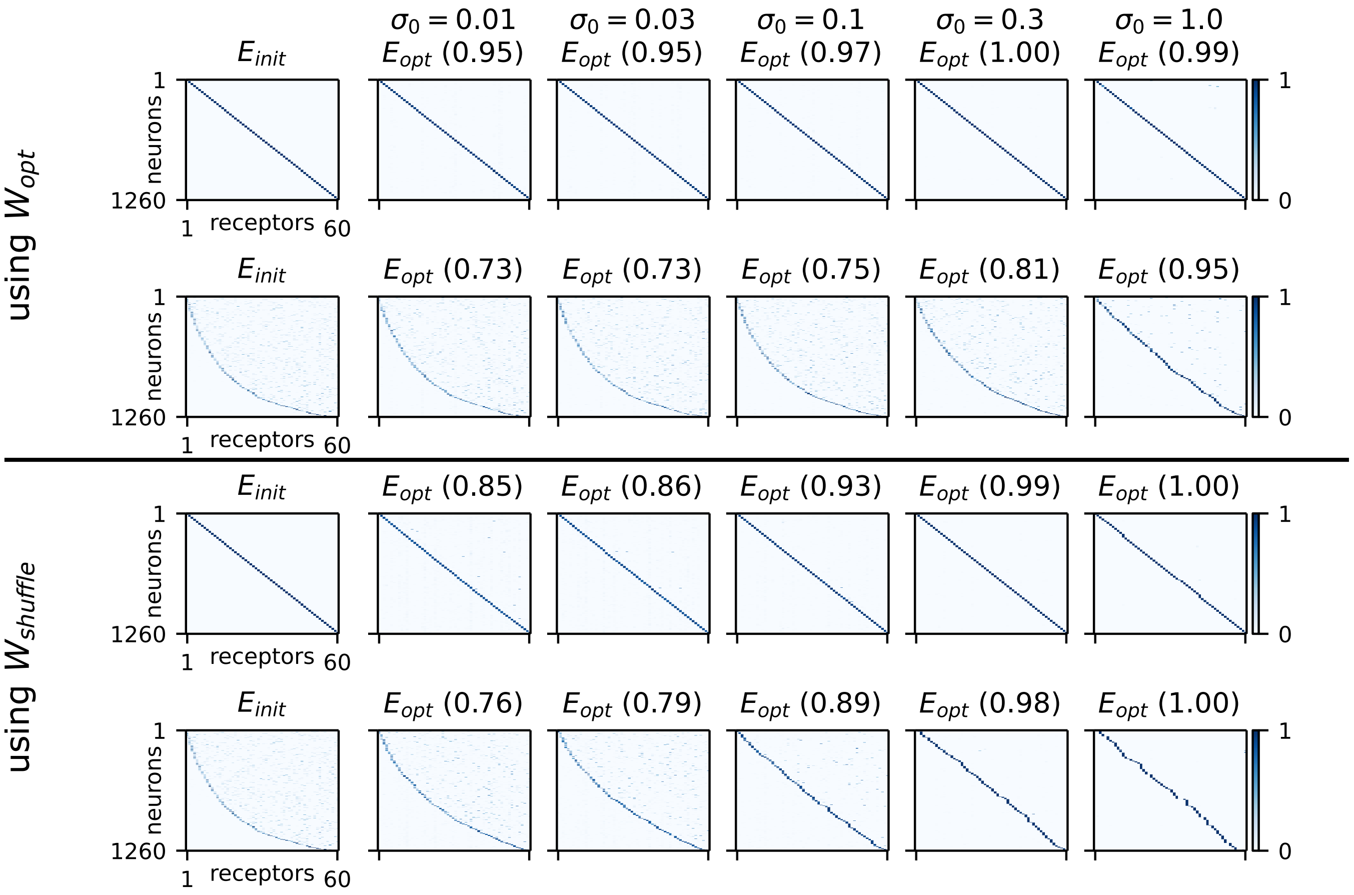

FIG. S12. Sweeping across neural noise σ_0. The canonical score (as defined in Figure 5) is shown in parentheses. Both canonical and noncanonical initializations are shown. For very low levels of neural noise, some noncanonical expression is tolerated. For $\sigma_0 \in [0.1, 1.0]$, canonical olfaction is strongly favored. Environmental parameters are set to 64 sources and $\sigma_c = 2$.

bioRxiv preprint doi: https://doi.org/10.1101/2025.09.03.673748; this version posted September 3, 2025. The copyright holder for this preprint (which was not certified by peer review) is the author/funder, who has granted bioRxiv a license to display the preprint in perpetuity. It is made available under aCC-BY 4.0 International license.

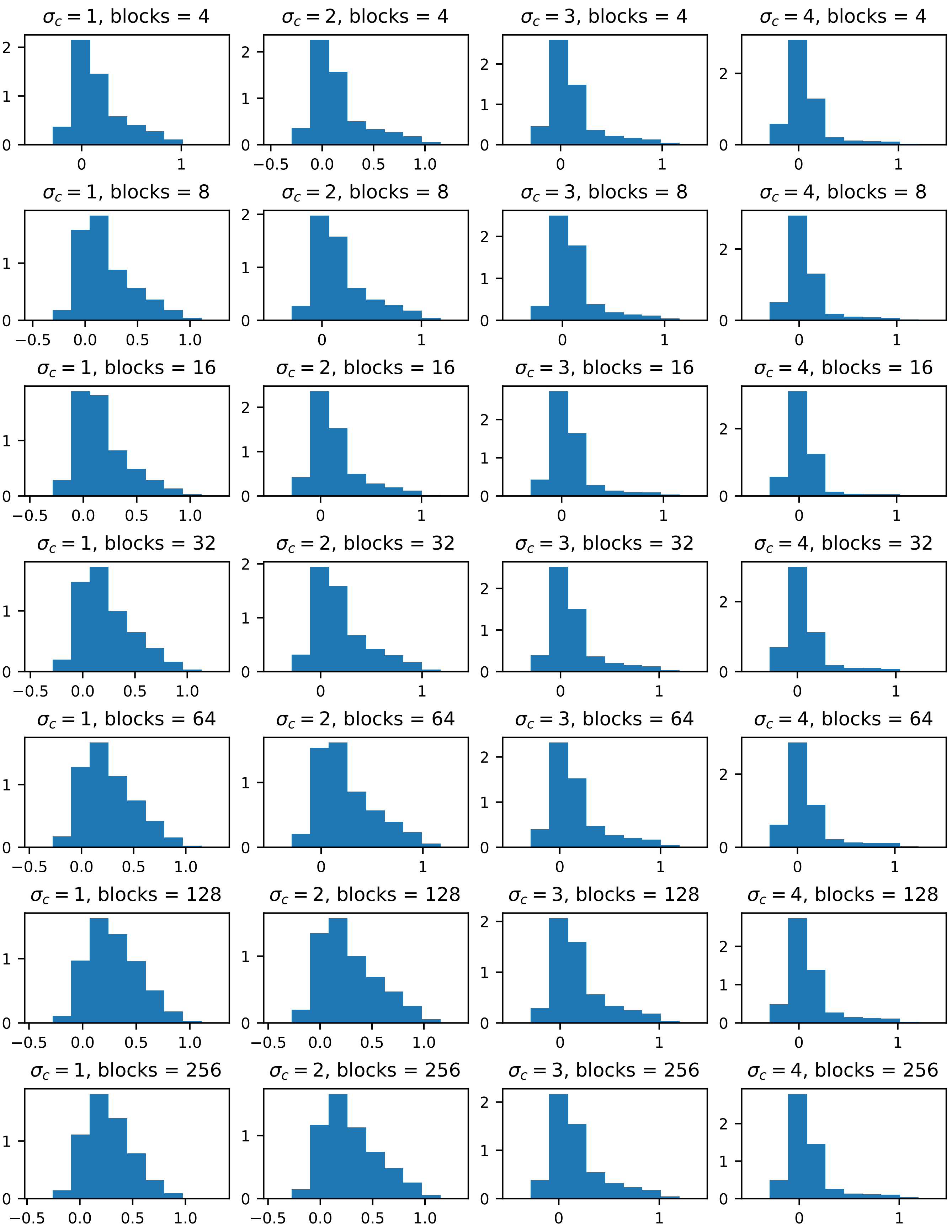

FIG. S13. ORN activity distributions across environmental parameters using shuffled W and corresponding optimized E.

bioRxiv preprint doi: https://doi.org/10.1101/2025.09.03.673748; this version posted September 3, 2025. The copyright holder for this preprint (which was not certified by peer review) is the author/funder, who has granted bioRxiv a license to display the preprint in perpetuity. It is made available under aCC-BY 4.0 International license.

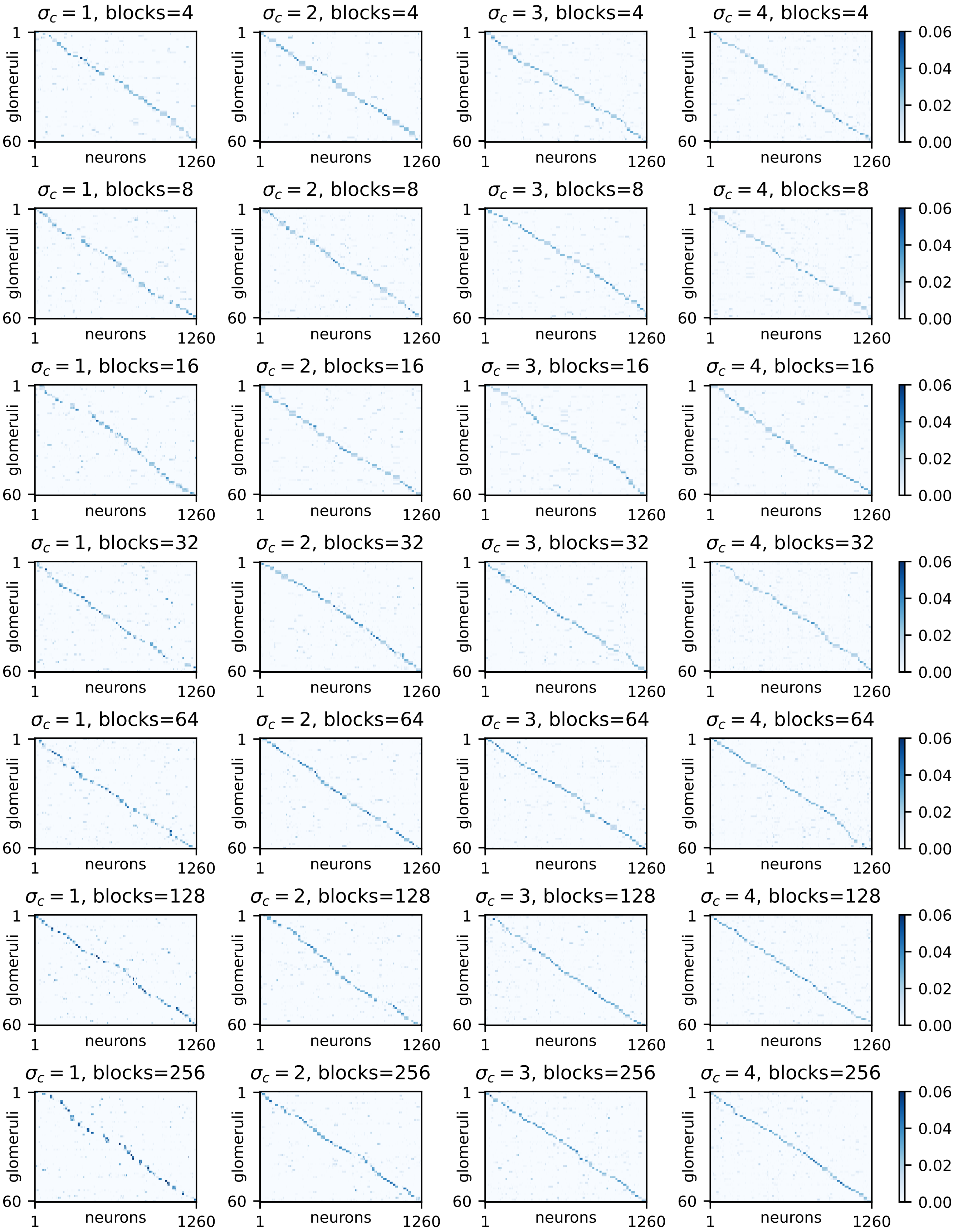

FIG. S14. Glomerular convergence across environments using shuffled W and corresponding optimized E.

bioRxiv preprint doi: https://doi.org/10.1101/2025.09.03.673748; this version posted September 3, 2025. The copyright holder for this preprint (which was not certified by peer review) is the author/funder, who has granted bioRxiv a license to display the preprint in perpetuity. It is made available under aCC-BY 4.0 International license.

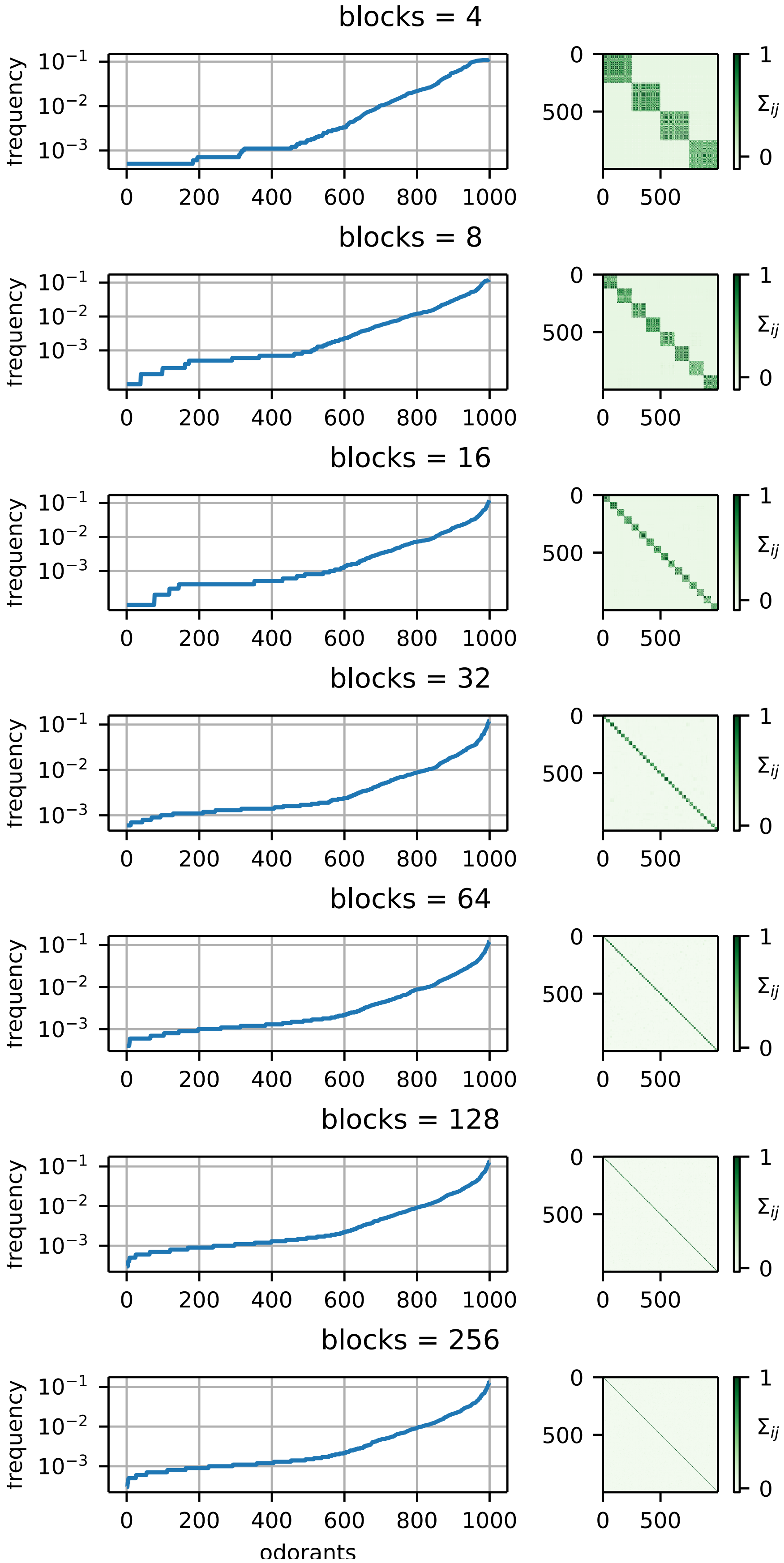

FIG. S15. Mean and covariance of the binarized odorant vector c_{bin} as the number of blocks k is varied.
